# Baselines Matter: Mass Spectrometric Assessments of Biological O_2_ Supersaturation (ΔO_2_:Ar) Benefit from Two-Point Calibrations

**DOI:** 10.1021/acs.analchem.5c02252

**Published:** 2025-11-25

**Authors:** Sebastian D. Rokitta, Emelia J. Chamberlain, Alessandra D′Angelo, Jeff S. Bowman, Brice Loose, Adam Ulfsbo, Allison A. Fong, Klaus-Uwe Richter, Sven A. Kranz, Björn Rost

**Affiliations:** † 9151 Alfred-Wegener-Institute—Helmholtz-Centre for Polar and Marine Research, 27570 Bremerhaven, Germany; ‡ 10627 Woods Hole Oceanographic Institution, Woods Hole, Massachusetts 02543, United States; § 4260 University of Rhode Island, Graduate School of Oceanography, Kingston, Rhode Island 02881, United States; ∥ 8784 University of California San Diego, La Jolla, California 92093, United States; ⊥ 3570 University of Gothenburg, Department of Marine Sciences, 40530 Gothenburg, Sweden; # 3990 Rice University, Houston, Texas 77005, United States; ∇ University of Bremen, Department of Biology & Chemistry, 28359 Bremen, Germany

## Abstract

Membrane-inlet mass spectrometry (MIMS) based assessments of ΔO_2_:Ar to estimate marine productivity are becoming a widely used tool in biogeochemistry. Especially continuous ship-borne surveys of dissolved gases allow for high spatial and temporal resolution in the analysis of surface ocean net community productivity. Depending on instrument configuration and architecture, however, measurements may be afflicted with substantial detection baselines for each analyzed gas. We hypothesized that ignoring these baselines (as seems to be common practice) can considerably affect the outcomes of the measurements. Using MIMS data from two cruises, we assessed the impact of calibration procedures and different pressure regimes, i.e., gas loads, on the ΔO_2_:Ar ratios and analyses of net community productivity. We compared conventional ratio-based one-point calibration approaches with two-point calibration approaches that include baselines and either calibrate for the equilibrium ratio of O_2_:Ar, or calibrate for the individual gas concentrations. Our data show that higher gas loads increase apparent signal heights, but also disproportionately increase the individual gas-associated baseline levels, thereby reducing instrument sensitivity while increasing a potential bias if data is used without correction. Depending on the adjusted gas loads, severe underestimations of final ΔO_2_:Ar values by factors of ca. 1.4 and 4 were calculated when disregarding baselines, emphasizing the critical importance of baseline determinations in MIMS-based methods.

## Graphical abstract

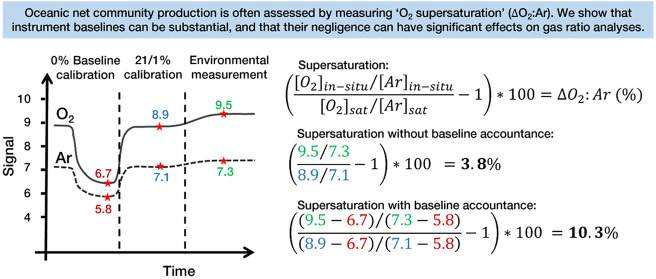

## Introduction

Mass spectrometric measurements of the ratio of oceanic Oxygen (O_2_) and Argon (Ar) are becoming a widely applied tool in biogeochemical research to estimate local and regional net community productivity (NCP) and draw conclusions regarding ecosystem functioning.^1–5^ The approach is based on the premise that the biologically induced O_2_ super- or undersaturation (i.e., positive or negative ΔO_2_:Ar) is an integrative record of the relative intensities of oxygenic photosynthesis and community respiration, while accounting for vertical mixing and air-sea gas exchange in the investigated water parcel for time scales of the gas residence time (10–14 days^2,6,7^ ). The overall O_2_ super- or undersaturation, comprising physical and biological components, is classically defined as the ratio of in situ O_2_ concentration to the theoretical equilibrium concentration at a given temperature and salinity (T, S). It is, however, challenging to quantify the physical and biological components independently. To disentangle the physical and biological components of the O_2_ saturation state, mass spectrometric methods exploit the essentially identical solubility properties of O_2_ and Ar in seawater:^8^ In a system without biology, physical changes (e.g., in T or S) will cause the same degree of super- or undersaturation in the two gases, resulting in a constant ratio of fugacities and equilibrium concentrations. Once biology is included, O_2_ is produced and consumed via processes like photosynthesis and respiration, resulting in excursions from the abiotic O_2_:Ar equilibrium ratio. Since mass spectrometers are very precise, especially in the detection of mass ratios, the MIMS method is considered to be extremely sensitive in the determination of the biological O_2_ supersaturation. As an example, at 1 °C (high latitude oceans) and a salinity of 32, a biologically caused O_2_ excess or deficit of only 1 μmol kg^–1^ above or below the equilibrated background of 347 μmol kg^–1^ (i.e., 0.3% deviation; Ar = ∼17 μmol kg^–1^) would cause the O_2_:Ar ratio to substantially shift from 20.44 up to 20.59 or down to 20.38, respectively (Figure 1; References 7–10 ).

**Figure 1:**
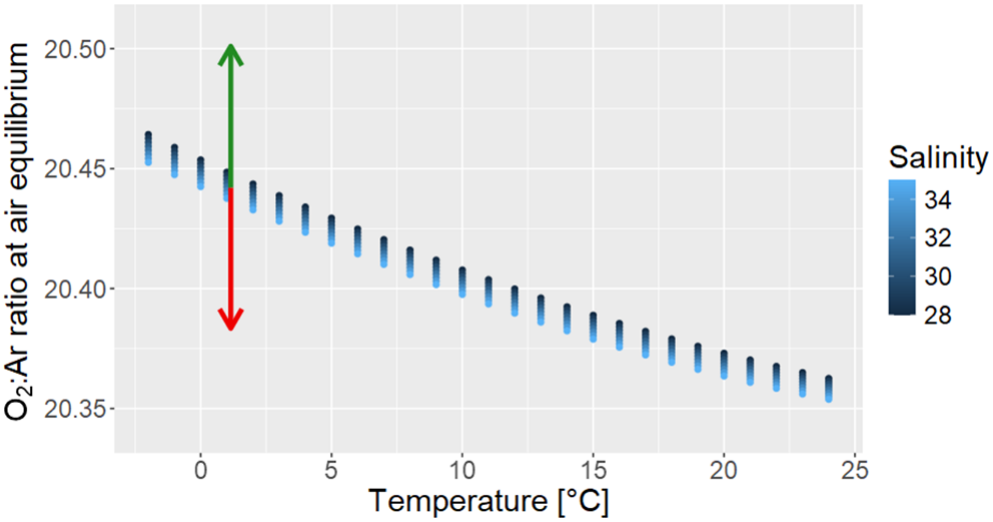
Range of O_2_:Ar equilibrium ratios in seawater (blue dots) across gradients of salinity (28 to 35, dark blue to light blue) and temperature (−2 to 24 °C, *x* axis), showing that the O_2_:Ar ratio in seawater at air equilibrium is practically constant at ∼20.4. Arrows indicate the excursion of the ratio at a given biological O_2_ production (green) or consumption (red) of 1 μmol kg^–1^.

Figure 1 also indicates that temperature shifts exceeding 10 °C would be necessary to produce errors in biological O_2_ budget estimates greater than 1 μmol kg^–1^. The effect of salinity (gradient from dark blue to light blue in Figure 1) on the equilibrium ratio is even less pronounced, suggesting that the method remains effectively robust against variations or inaccuracies in seawater salinities.

The parameter “biologically induced deviation from O_2_ saturation”, ΔO_2_:Ar, (positive values denote supersaturation, negative values denote undersaturation) principally compares the observed in situ concentration ratio of O_2_ and Ar to the concentration ratio obtained (or expected^12,13^ ) at air-equilibrium (eq 1)­

1 $$\Delta O_{2}:\mathrm{Ar}\;\left(\%\right)=\left[\frac{{{[O}_{2}]}_{in‐situ}/{\left[\mathrm{Ar}\right]}_{in‐situ}}{{{[O}_{2}]}_{sat}/{\left[\mathrm{Ar}\right]}_{sat}}-1\right]\times 100$$

This supersaturation can be used to derive estimates of NCP (eq 2; e.g., Ulfsbo et al.^14^)­

2 $$\mathrm{NCP}\approx k_{O_{2}}\times {\left[O_{2}\right]}_{\mathrm{sat}}\times \Delta O_{2}:\mathrm{Ar}\times 1/\mathrm{PQ}$$

where *k*_O_2__ is the gas transfer or ‘piston’ velocity for O_2_ (m d^–1^), [O_2_]_sat_ is the O_2_ equilibrium concentration in seawater,^9,10^ and PQ is the photosynthetic quotient, which estimates photosynthetically fixed carbon from the concomitantly generated O_2_.^15^ The outlined approach appears quite straightforward at first, but a number of approximations and assumptions need to be made. The gas transfer velocity between ocean and atmosphere, for instance, is among the most difficult parameters to constrain. This parameter strongly depends on surface wind speed,^16,17^ but phenomena like surface wave motion and ripple, bubble entrainment, presence of surfactants, mixed layer depth and internal waves,^18–21^ which are extremely difficult to quantify, have been shown to play major roles modulating the gas transfer. The photosynthetic quotient PQ, i.e., the molar ratio of O_2_ produced per CO_2_ assimilated during photosynthesis, is a parameter that is typically used like a constant (e.g., fixed PQ of 1.4) for all phototrophs through time and space,^14,15^ whereas recent research indicates that it can not only vary widely^22^ (between 0.1 and 4.2) but also rapidly^23^ (minutes) depending on environmental settings. Aside from these considerations, a negative ΔO_2_:Ar indicates that respiratory processes dominate the water parcel, calling for the use of a respiratory quotient rather than a photosynthetic quotient. It becomes clear that more understanding and conceptualization is needed to further improve estimates of CO_2_ dynamics at the ocean-atmosphere interface derived from surface O_2_ and Ar concentrations.

A range of papers about gas transfer velocity derivations, O_2_ residence time and effects of physics on O_2_:Ar ratios exists,^19,20,24^ but potential biases from MS configurations, vacuum system architecture and calibration techniques are rarely discussed. In most oceanographic studies, for instance, mass spectrometers are typically “one-point ratio calibrated” by directly measuring air or air-equilibrated water and building the ratio of O_2_:Ar as a reference calibrator.^2,13,25,26^ Only very few studies, often focused on trace gas analyses, employ more complex multipoint calibrations.^4,27^ This widespread use of "one-point ratio calibrations" seems to be the acknowledged consensus and common practice in the oceanographic community, but will be critically assessed here.

First of all, “ratio calibrations”, in which the ratios of raw mass signals rather than the individual gas concentrations are used for calibration do not allow reconstruction of the true abundances of the single gases. Since O_2_:Ar ratios can vary due to the changes in the numerator value, the denominator value or both, important information on concentrations of O_2_ and Ar is missing if an instrument is only ratio-calibrated to the equilibrium point. One approach commonly taken in an attempt to estimate O_2_ concentrations from ratio-calibrated MS measurements is to assume Ar equilibrium concentrations^28^ so that [O_2_]_sat_ × (1 + ΔO_2_:Ar) translates to an approximate O_2_ concentration. However, this approach has been challenged by the recognition that Ar is seldomly close to perfect equilibrium.^29,30^

A second shortcoming of one-point ratio calibrations is that, strictly speaking, it is technically invalid to apply the obtained ratio-calibrators to O_2_ and Ar signal ranges that differ from those used during the instrument calibration. The reason for this is that even if an “ideal” mass spectrometer behaves perfectly linear for each single gas over their entire measuring range, it would be virtually impossible that the linearities (i.e., calibration factors) are the same for the two compared gases (Figure 2). This phenomenon derives from different membrane permeabilities and ionization susceptibilities of analytes, and can also be easily seen in the fact that the observed ratio of raw traces of O_2_ and Ar, e.g., during calibration with air-equilibrated water (e.g., ∼11 in our instrument, ∼12 in reference 22), does not necessarily resemble the real molar ratio of gas concentrations in the environment (which is ∼20.4, Figure 1). EIMS instruments, that do not involve membrane permeation, in contrast, seem to yield calibration ratios more close to the molar ratio of these gases in air.^2^

**Figure 2:**
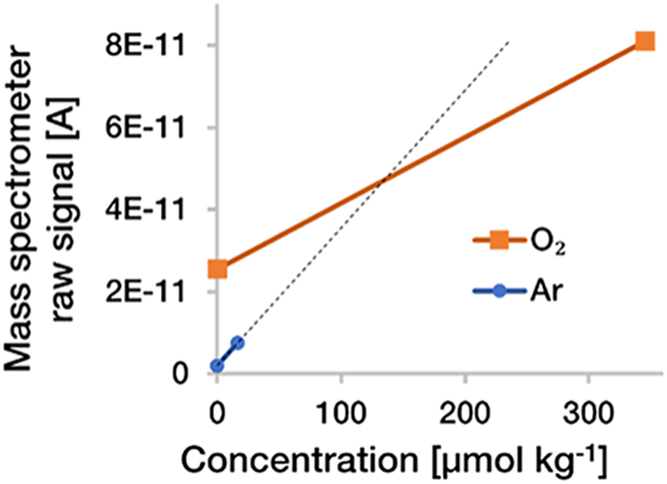
Exemplary two-point calibrations of O_2_ and Ar obtained using our here described MIMS system. Linear interpolation between the two calibration points shows that the instrument has different responsiveness for O_2_ and Ar; The slope for Ar is more than two times that of O_2_ indicating a higher Ar permeability of the membrane used.

As a consequence of the two different calibration slopes (Figure 2), using a one-point ratio calibration would lead to overestimations of ΔO_2_:Ar at O_2_ and Ar concentrations below the calibration concentrations, and to underestimations of ΔO_2_:Ar, when O_2_ and Ar concentrations are above the calibration concentrations. Especially when the gas loads and pressures measured in the mass spectrometer during calibration are very different from the situation of sample measurements, e.g., when calibrating with air while measuring in a water stream, this phenomenon might significantly impede measurements.

We hypothesize that a solution for these shortcomings could be the use of two-point calibrations that add the instrument baseline measurements obtained by measuring O_2_ and Ar signals in Nitrogen (N_2_) gas purged (0% O_2_ and 0% Ar) calibration samples. N_2_ gas, or water purged with N_2_ sufficiently to drive out O_2_ and Ar, can be sampled and the recorded signals can serve as baseline detection (i.e., [O_2_] and [Ar] = ∼0 μmol kg^–1^). The advantages appear significant: Two-point calibrations principally allow the explicit and independent determination of O_2_ and Ar concentrations, as well as the calculation of their respective supersaturations compared to air-equilibrium. In the case of the biologically inaccessible Ar, its supersaturation directly reflects physical phenomena, enabling the full numeric disentanglement of the physical and the biological components of the O_2_ signal in the water.^6^

In this study, we therefore seek to quantify uncertainties and biases that may arise from different instrument configurations and calibration practices. To this end, we use exemplary data sets from two cruises where our MIMS was operated at different gas loads, and we directly compare the utility of conventional “one-point ratio calibrations” (1R) with “two-point ratio calibrations” (2R) as well as “independent two-point calibrations” (2I) of O_2_ and Ar, respectively.

## Material and Methods

We present data excerpts from two cruises, the MOSAiC and the ISLAND IMPACT cruise. The MOSAiC cruise was a year-long drift study in 2019/2020, in which the research vessel POLARSTERN was moored to an ice floe in the high Arctic. A multidisciplinary scientific observatory was established on the floe, and in an unprecedented logistic effort, chemical, meteorological and biological data were collected through large portions of the polar night.^4,31,32^ The second data set derives from the ISLAND IMPACT cruise, in which POLARSTERN went to the Southern Ocean (SO), specifically South Georgia and the South Sandwich Islands in 2022, to study the influence of trace metals leached from the island on the regional primary productivity and carbon export (Klaas et al. in prep.).

In both cruises, our custom-built MIMS system (Figure 3a) was set up to monitor O_2_ and Ar in the surface ocean, represented by masses 32 and 40 detected in the mass spectrometer (Pfeiffer QMG220 Prisma Plus quadrupole with Faraday detector). The mass spectrometer is attached to the evacuated vacuum system (pressure at ion source during measurements was ca. 5 × 10^–6^ to 8 × 10^–7^ mbar), which couples to a custom-built seawater flow-through cuvette (Figure 3b). The membrane inlet cuvette is housed in a 4-way cross junction compatible with the rest of the tubing used in the system (PTFE material, 6 mm outer diameter). Horizontally, a hollow PTFE support rod with a recess is inserted, holding the membrane inlet, i.e., a stainless-steel vacuum capillary in which 150 holes were shot with an ablation laser (surface crater size 30 μm, final hole size 10 μm). The capillary is covered with a silicone tubing (0.1 mm thickness). The terminal end of the capillary is welded to ensure gas tightness. Gases in the water dissolve into the membrane and then transition to the vacuum, following the steep pressure gradient. They are passed along a modified pulse-tube cooler (PXE 100, AIM Germany) that exposes a 170 cm^2^ metal surface of −95 °C to the vacuum that traps water vapor in order to enhance the sensitivity of the instrument (see Results section).

**Figure 3:**
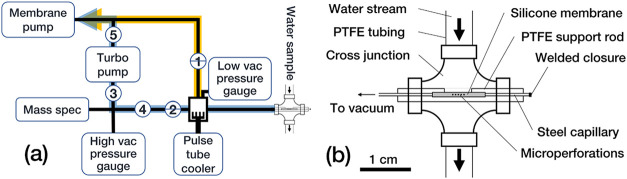
(a) Schematic layout of the used MIMS System. Vacuum lines (black) connect the membrane inlet (right) to the mass spectrometer (left) while conducting all gases along the pulse tube cooler for cryo-distillation. Reciprocal switchover valves (1, 2) direct the gas flux either along the mass spectrometer (lower branch, blue arrow), or circumvent the mass spectrometer and the turbo pump (upper branch, yellow arrow). Manual gate and gas leak valves (3 and 4) are implemented to fine-tune pressure gradients and gas delivery between the membrane and the mass spectrometer ion source. When the pulse tube cooler needs to be defrosted, gases are conducted via the upper branch, and an isolation valve (5) is closed to protect the turbo pump and MS from abrupt downstream pressure bursts. (b) Schematic of the custom-built membrane inlet cuvette.

To investigate the influence of gas load on baselines, adjustments on the instrument′s valves were made for the ISLAND IMPACT cruise, in order to allow higher gas delivery to the ion source. Specifically, we opened the gas leak valve (4 in Figure 3a), causing lower pressure at the membrane and allowing higher gas intake into the vacuum. With more gas intake, also the pressure at the source increased, which was balanced by opening the manual gate valve (3 in Figure 3a) allowing the turbo pump to bring the source pressure to the desired operating range. This distinct feature between the two cruise data sets drew our attention to the impact of signal intensity, i.e., the overall gas load, on instrument performance, prompting us to investigate this effect more closely.

The equilibrator unit (Figure 4) was continuously fed by the ship′s underway sampling system that delivers water from the keel intake at ∼11 m depth into the lab. The Teflon-lined pipes of the ship are regularly cleaned with bleach during port-maintenance, to counteract signal distortion due to biological activity as good as possible.^33^ The flow of water delivered to the equilibrator was chosen to be high to additionally ensure a low residence time of water in the pipes (estimated to be <4 min). To counteract biofilm formation and biological activity within our instrument over time, incoming underway seawater was kept dark and was prefiltered through a 100 μm mesh. The mesh filter was changed every day to counteract the accumulation of planktonic organisms that could affect downstream gas measurements. In regular intervals of 1–2 weeks, the gas equilibrator and inlet system were disassembled, and thoroughly washed with an alkaline detergent (MUCASOL) that does not liberate gases (as would be the case with hypochlorite, hydrogen peroxide, or acids).

**Figure 4:**
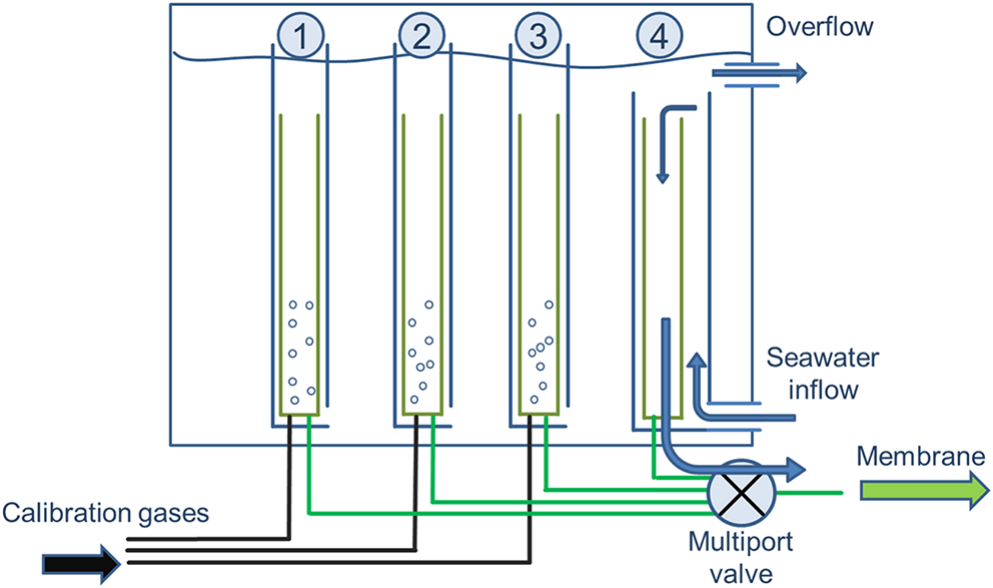
Schematic of the used equilibrator. Seawater is fed into the equilibrator (right), directly enters the underway sampling cylinder (#4, green), and is conducted to the membrane via an outlet multiport valve at the bottom of the cylinder (green). Excess water will overflow the sampling cylinder, successively fill the calibration cylinders (#1–3, green) and then the rest of the equilibrator, until the water level reaches the overflow. This setup assures that always the “newest” seawater is entering the underway sampling cylinder, that excess water is concomitantly used as coolant and that the hydrostatic pressure driving the water flow at the membrane remains constant. The calibration cylinders can be purged with desired calibration gases (left), and after sufficient time, valves can be switched to sample the desired calibration solution.

Calibrations were performed in regular intervals (24–72 h) to monitor drifting baselines and instrument dynamic range over the cruises. Instrument baselines for O_2_ and Ar (“0% calibrations”) were obtained by measuring seawater that was purged with N_2_ gas (Air Liquide ALPHAGAZ 1, ≥99.999%) for at least 30 min inside the equilibration cylinders (Vigreux distillation columns, to enhance the path length for the rising gas bubbles and thus, gas transfer). Air-equilibrium abundances for O_2_ and Ar (“21/1% calibrations”) were obtained by measuring seawater purged with compressed air (Air Liquide ALPHAGAZ 1) for at least 30 min. After the equilibration time, the seawater within the calibration cylinders was successively sampled before switching back to underway sampling mode. During development of the instrument, we verified that the gas purging was sufficient in intensity and duration, as was evident by the fact that the purged samples achieved practically the same baselines as observed after the addition of sodium dithionite, a chemical that chemically strips oxygen.

For a general description of the performance and the utility of the cryo distillation, the sensitivity (eq 3) and responsiveness of the instrument for O_2_ and Ar was exemplarily determined in the lab by measuring two known concentrations (i.e., 0 and 275 μmol kg^–1^ O_2_, which were obtained by sufficiently purging deionized water with N_2_, and by equilibrating deionized water at room temperature, respectively). Dividing this concentration difference by the fraction of the difference in signal height over the signal noise (here we conservatively used the difference between the highest data point and the lowest data point within a ±15 s time window around calibration time points) yields the sensitivity of the instrument, i.e., the smallest detectable signal change.^34^

3 $$\mathrm{sensitivity}\;\left[{\mu \mathrm{mol}\;\mathrm{kg}}^{-1}\right]=\frac{\mathrm{concentration}\;\mathrm{difference}\;\left[\mu \mathrm{mol}\;{\mathrm{kg}}^{-1}\right]}{(\mathrm{signal}\;\mathrm{difference}/\mathrm{signal}\;\mathrm{noise})}$$

All MIMS data were recorded in time resolutions <5 s. Auxiliary high-resolution data including simultaneously recorded temperatures and salinities from the ship′s thermosalinograph (SeaBird SBE21 with integrated SBE38 temperature sensor) as well as position and other environmental data were retrieved from the ship ´s logging systems and were amended to the data set. When time stamps of auxiliary data mismatched with those of MIMS data, said auxiliary data were linearly interpolated for the respective time stamps. Afterward, data without MIMS measurements were removed to preserve “tidy”, i.e., gapless data. Lastly, the data set was time averaged using the *openair* package^35^ for R,^36^ bringing periods of underway data to 1h resolution, and periods of calibration data to 1 min resolution. In all considerations mentioned, the O_2_ and Ar equilibrium concentrations as well as respective ratios were calculated based on the corrected formula of Garcia and Gordon^9,10^ as implemented in the *gsw* package^37^ for R as well as the equations by Hamme and Emerson.^11^

To obtain the conventional “one-point ratio calibrated” biological supersaturation, ΔO_2_:Ar_(1R)_ (eq 4), any determined baselines were intentionally disregarded. To obtain “moving” calibrators for every time point, O_2_:Ar ratios measured at 21/1% calibration time points were linearly interpolated between the determinations, and the outermost calibrator values were extended to the very start and end of the data set. To obtain the "two-point ratio calibrated" biological supersaturation, ΔO_2_:Ar_(2R)_ (eq 5), quality-checked 0% baseline determinations for O_2_ and Ar were linearly interpolated between determinations and the outermost baseline values were extended to the ends of the data set. The baseline was then subtracted from the raw data trace prior to the determination of the “moving” calibration from the corrected M32/M40 ratio as outlined above. To obtain the “two-point independently calibrated” biological supersaturation ΔO_2_:Ar_(2I)_ (eq 6), the traces of O_2_ and Ar were first baseline corrected as outlined above. For the air-equilibrium points, we calculated the theoretical equilibrium concentrations of O_2_ and Ar achieved in our equilibrator based on seawater salinity and temperature measured within the flow cuvette (High-precision Pt100 thermoprobe, precision ± 0.05 °C). Subsequently, a linear calibration factor was calculated that (before calibration) translates between the baseline-corrected signal and the gas concentration in μmol kg^–1^ (eq 7, square brackets indicate concentrations, not mass signals) at the 21/1% calibration time points. Moving calibrators were obtained by linear interpolation, and the outermost calibrators were extended to the start and end of the data set, which enabled the independent calculation of O_2_ and Ar concentrations, as well as the resulting ΔO_2_:Ar_(2I)_ by directly dividing the obtained gas concentrations. A flowchart of data processing is given in Supporting Figure SF1.

4 $${\Delta O_{2}:Ar}_{\left(1R\right)}\;\left(\%\right)=\left(\frac{{O_{2}}_{raw;in‐situ}/{Ar}_{raw;in‐situ}}{{O_{2}}_{raw;equilibrium}/{Ar}_{raw;equilibrium}}-1\right)\times 100$$

5 $${\Delta O_{2}:\mathrm{Ar}}_{\left(2R\right)}\;\left(\%\right)=\left(\frac{({O_{2}}_{\mathrm{raw};\mathrm{in}‐\mathrm{situ}}-{O_{2}}_{\mathrm{baseline}})/\left({\mathrm{Ar}}_{\mathrm{raw};\mathrm{in}‐\mathrm{situ}}-{\mathrm{Ar}}_{\mathrm{baseline}}\right)}{\left({O_{2}}_{\mathrm{raw};\mathrm{equil.}}-{O_{2}}_{\mathrm{baseline}}\right)/\left({\mathrm{Ar}}_{\mathrm{raw};\mathrm{equil.}}-{\mathrm{Ar}}_{\mathrm{baseline}}\right)}-1\right)\times 100$$

6 $${\Delta O_{2}:\mathrm{Ar}}_{\left(2I\right)}\;\left(\%\right)=\left(\frac{{\left[O_{2}\right]}_{\mathrm{in}‐\mathrm{situ}}/{\left[\mathrm{Ar}\right]}_{\mathrm{in}‐\mathrm{situ}}}{{\left[O_{2}\right]}_{\mathrm{equilibrium}}/{\left[\mathrm{Ar}\right]}_{\mathrm{equilibrium}}}-1\right)\times 100$$

whereas

7 $${\left[\mathrm{Gas}\right]}_{\mathrm{in}‐\mathrm{situ}}=\left(\frac{\mathrm{Gas}\;\mathrm{Eq}.\mathrm{conc}.}{\left({\mathrm{Gas}}_{\mathrm{equlibrium}}-{\mathrm{Gas}}_{\mathrm{baseline}}\right)}\right)\times \left({\mathrm{Gas}}_{\mathrm{raw}}-{\mathrm{Gas}}_{\mathrm{baseline}}\right)$$

## Results

Since our instrument is custom-built and operates an uncommon cryo distillation stage, we have determined the instrument′s sensitivity and the effectiveness of the cryo distillation module in an experiment that highlights the influence on the instrument′s gas load. To this end, we present two calibrations done in the lab with deionized water at room temperature, first without and then with the cryo-distillation module in operation (Figure 5; 13:30–14:10h vs 14:15–14:40h). The effectiveness of the cryo-distillation is recognizable by the raw signal of mass 18 (i.e., H_2_O) dropping to merely 6% of the original intensity, from approximately 1.4 × 10^–9^ to ca. 0.9 × 10^–10^ when the temperature gets lower than the sublimation point at the system vacuum (14:10h). A simultaneous increase in the masses 32 (O_2_) and 40 (Ar) was observed as direct response while keeping the air equilibrated water flow constant.

**Figure 5:**
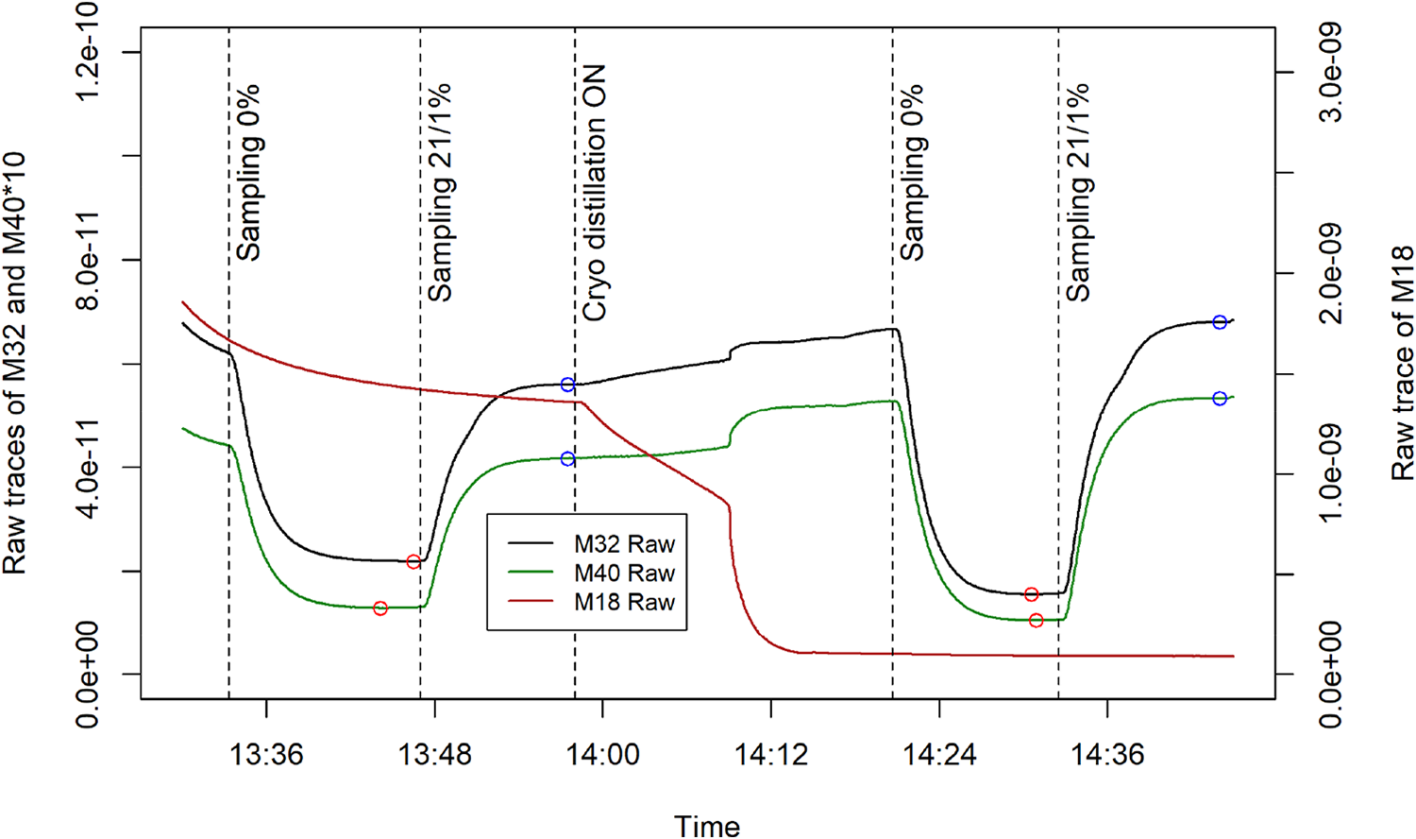
Two calibrations in deionized water, done without (left) and with (right) cryo distillation. Points mark baseline (red) 0% and topline (blue) 21/1% determinations, vertical dashed lines indicate sample switches for orientation.

Determined sensitivities (eq 3) for O_2_ and Ar without cryo-distillation were 0.19 and 0.04 μmol kg^–1^, respectively. With cryo-distillation enabled, lower baselines and higher toplines were achieved in the calibration, improving O_2_ and Ar detection sensitivities to 0.12 and 0.015 μmol kg^–1^, respectively. Typical “Tau_60_” response times, i.e., the duration until 60% of a defined signal step have been accomplished, were ca. 5–7 min for the two gases and were not affected by cryo-distillation. In field conditions, i.e., low water temperatures, steady signal plateaus are typically reached with the instrument within 15–25 min, depending on the temperature of sample and membrane (See also Supporting Figure SF2 for exemplary calibrations from the cruises)

The data shown from the two cruises represent overall traveled distances of ca. 1600 and 6000 km, respectively (Figure 6a,b). During the Arctic cruise, water temperatures were consistently at about −1.7 °C, and salinity experienced some small longer-term excursions, reflecting periods of sea ice formation during the months-long drift^38^ (Figure 6c). During the SO cruise (Figure 6d), temperatures were generally higher due to the temperate/subpolar setting, and the transit through water masses is well visible from temperature and salinity patterns (Oct 11–13: From the Antarctic Circumpolar Current into the South Atlantic Current and back; Oct 21st and 23rd: From the ACC into the Antarctic Subpolar Zone and back). Due to the valve adjustments, the source pressure (i.e., overall gas load) and consequently also the recorded raw signals were ca. 50% higher during the SO cruise compared to the Arctic cruise (Figure 6e,f and Table 1). At lower source pressure during the Arctic cruise, the baselines of O_2_ and Ar (determined with N_2_ purged seawater) during measurement contributed ca. 29 and 25% to the raw signal, respectively, whereas at higher source pressure in the SO cruise, baselines of O_2_ and Ar contributed ca. 76 and 73% to the raw equilibrium signals, respectively (Figure 6e,f and Table 1). After baseline subtraction, the remaining dynamic range of the recorded data was approximately 2-fold higher during the Arctic cruise (Figure 6g,h and Table 1) than during the SO cruise. The calibration factors determined for O_2_ and Ar during the Arctic cruise (required for the ΔO_2_:Ar_(2I)_ approach; Figure 6i) were approximately 6 and 3 × 10^12^ (μmol kg^–1^) A^–1^ for O_2_ and Ar, respectively, while during the SO cruise (Figure 6j), these calibration factors were approximately 50% higher for both gases. An overview of major operation parameters is given in Table 1.

**Figure 6:**
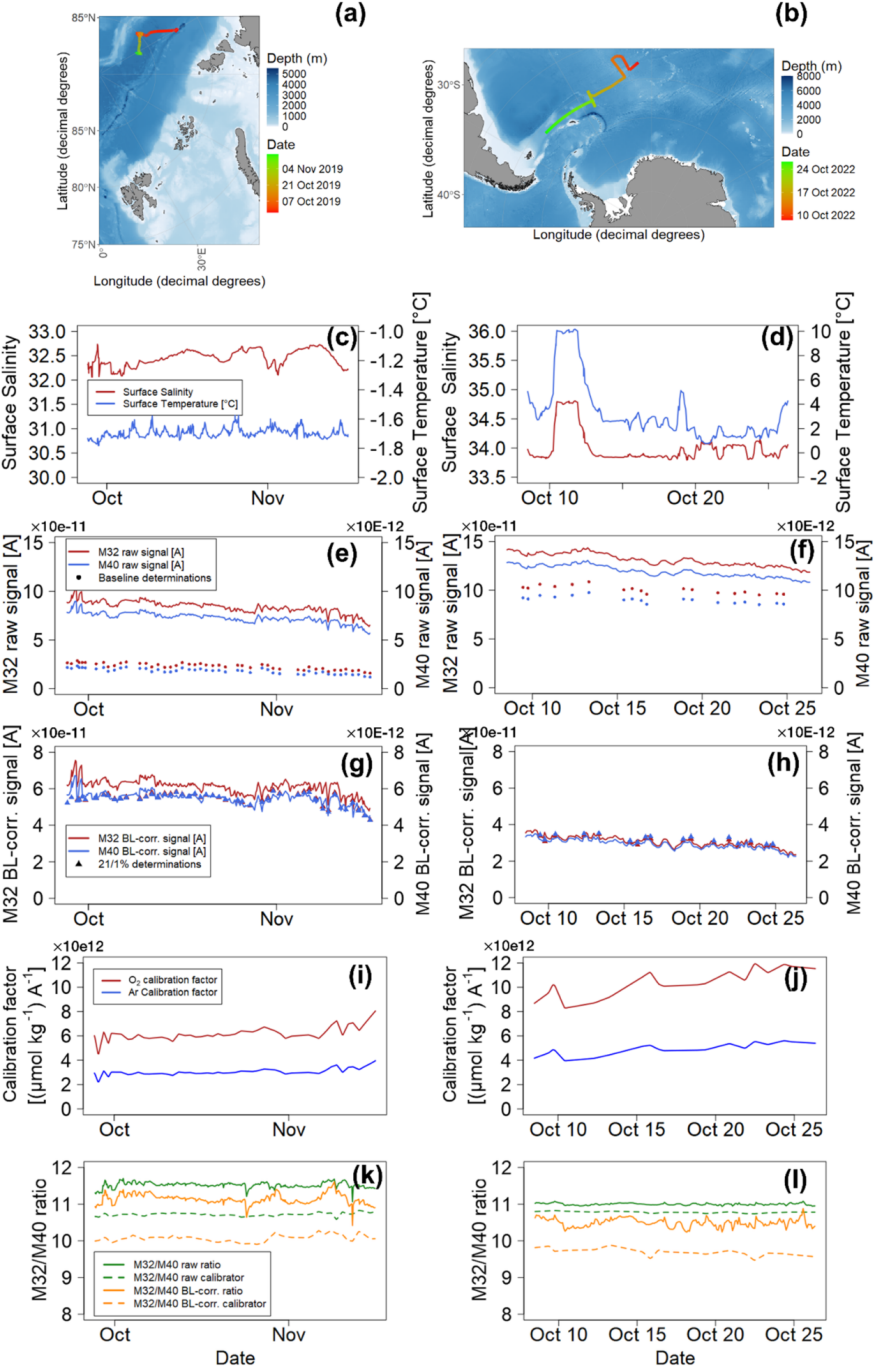
Cruise tracks and timing of the Arctic (a, left side) and Southern Ocean (b, right side) cruise; Temperature and salinity throughout the two cruises (c, d); Mass spectrometric raw data (e, f) of O_2_ (red) and Ar (blue) with respective baseline detections (points)­during the two cruises; Baseline-corrected data of O_2_ (red) and Ar (blue), as well as 21/1% determinations (triangles; g,h); Interpolated calibration factors for O_2_ (red) and Ar (blue) (i, j); raw (green) and corrected (orange) O_2_:Ar ratios of the obtained data (solid lines), as well as derived “moving” ratio calibrators (dashed lines; k, l).

**Table 1:** Major parameters of the MIMS operation during the two Cruises

parameter	arctic cruise PS122	southern ocean cruise PS133
average low vac pressure (mbar)	4.24 ± 1.5 × 10^–3^	2.04 ± 0.1 × 10^–3^
average source pressure (mbar)	9.0 ± 1.3× 10^–7^	3.1 ± 1.1 × 10^–6^
average O_2_ raw signal (A)	8.3 ± 0.6 × 10^–11^	1.31 ± 0.06 × 10^–10^
average Ar raw signal (A)	7.3 ± 0.5 × 10^–12^	1.2 ± 0.06 × 10^–11^
average O_2_:Ar raw ratio in data	11.52 ± 0.075	11.00 ± 0.027
average O_2_:Ar raw ratio at 21/1%	11.50 ± 0.60	10.97 ± 0.14
average O_2_ baseline:equilibrium raw signal	0.29 ± 0.03	0.76 ± 0.007
average Ar baseline:equilibrium raw signal	0.25 ± 0.02	0.73 ± 0.007
average O_2_ BL-corrected signal (A)	6.1 ± 0.4 × 10^–11^	3.1 ± 0.3 × 10^–11^
average Ar BL-corrected signal (A)	5.5 ± 0.3 × 10^–12^	2.9 ± 0.3 × 10^–12^
average O_2_:Ar BL-corrected ratio in data	11.14 ± 0.14	10.49 ± 0.11
average O_2_:Ar BL-corrected ratio at 21/1%	10.07 ± 0.09	9.69 ± 0.1
average O_2_ calibration factor ((μmol kg^–1^) A^–1^)	6.23 ± 0.53 × 10^12^	10.4 ± 1.1 × 10^12^
average Ar calibration factor ((μmol kg^–1^) A^–1^)	3.06 ± 0.26 × 10^12^	4.93 ± 0.51 × 10^12^
average O_2_ baseline drift (μmol d^–1^)	–1	–5
average Ar baseline drift (μmol d^–1^)	–0.09	–0.5

The O_2_ concentrations obtained from our two-point calibrated approach during both cruises (Figure 7a,b) were typically at or close to equilibrium and were overall in acceptable agreement (typically <10 μmol kg^–1^ deviation) with independent determinations^39^ using a Winkler-calibrated CTD mounted O_2_ sensor (SBE43, Seabird). The biological O_2_ supersaturation, ΔO_2_:Ar_1R_, determined with the conventional, uncorrected “one-point ratio approach” indicated biologically caused supersaturations of 3–10% during the Arctic cruise (Figure 7c) and 1–3% during the SO cruise (Figure. [Fig fig7]d). In contrast, the ‘two-point ratio calibrated’ approach and the “two-point independent concentration approach” (ΔO_2_:Ar_2R_ and ΔO_2_:Ar_2I_) showed biological supersaturations of ca. 4–14% during the Arctic cruise and 5–14% during the SO cruise (Figure 7c,d), and overall more pronounced dynamics in the data than obtained with the conventionally determined ΔO_2_:Ar_1R_. The results of the two baseline-corrected approaches were in very close agreement in both cruises, i.e., the ΔO_2_:Ar_2R_ approach yielded negligibly deviating biologically caused supersaturations (ca. 0.05 ± 0.024 percentage points) compared to the ΔO_2_:Ar_2I_ approach.

**Figure 7:**
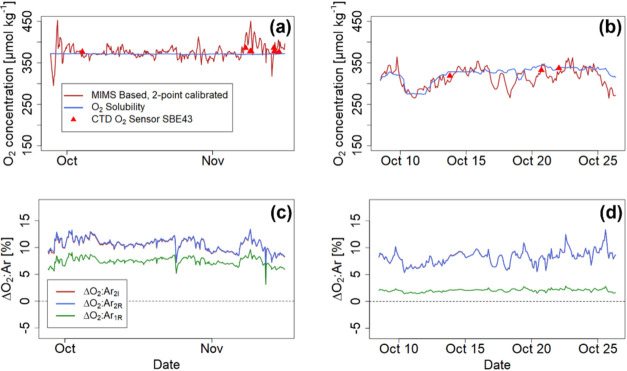
O_2_ concentrations observed during the Arctic (a) and SO cruises (b) as determined with the outlined two-point calibration; ΔO_2_:Ar values in the Arctic (c) and SO cruises (d) obtained with three different approaches.

## Discussion

The outlined exemplary performance assessments (Figure 5) show that our instrument allows a theoretical quantification of target analytes O_2_ and Ar in the lower nanomolar range and achieves response times of several minutes, comparable to other instruments.^2,40^ The cryo distillation effectively removes the majority of water vapor. This reduces the overall pressure, i.e., gas load in the vacuum system, which instantly lowers the baselines of our gases of interest and also improves the sensitivity of the analysis (Figure 5). There are multiple phenomena that contribute to this effect: First, Faraday detectors and their attached amplifiers will always exhibit an electronic baseline noise that is different from zero. Furthermore, these detectors integrate the impact of ions (of any kind), and thereby, they also detect so-called ‘scatter ions’ that randomly pass through the mass filter, even in the absence of a target analyte and target mass acceleration. Also, electron-stimulated desorption in the ion source as well as chemical recombination of analyte molecules can occur, which is known to give rise to molecules of the same mass as desired analytes.^41,42^ Lastly, in vacuum systems, surface ad/desorption of gases occurs, which causes elevated gas loads especially in vacuum system with increasingly complex geometry.^43–45^ These phenomena cause a variable baseline component (sometimes called “background”) that scales with operating pressure, i.e., gas load, and afflicts all investigated masses. The impact of increased background due to gas load is stronger for gases in lower concentration, e.g., Ar, as also shown in our measurements (Figure 5). In addition, with cryo distillation enabled, higher signals are observed for O_2_ and Ar during the 21/1% calibrations than without cryo distillation (Figure 5). This is because less water is present to compete for ionization, which increases the observed signals of target analytes. With this increased dynamic range (i.e., lower baselines, higher toplines), the cryo distillation stage improves sensitivity even further (by 65 and 180% for O_2_ and Ar, respectively). While this increase in sensitivity is not urgently required to measure O_2_ and Ar satisfyingly precise, a cryo-distillation stage can offer desirable advantages, especially for applications that detect trace analytes of low abundance or high reactivity (e.g., methane and certain other volatile organic compounds^46^). A trade-off, however, is the need to remove the trapped water by regularly defrosting the cryo-distillation module. This requires stopping the measurements and heating the freezing surface while redirecting the resulting gas burst to the membrane pump (cf. Figure 3a). The liberation of substantial amounts of water vapor and the subsequent removal prolongs maintenance and, importantly, requires personnel at the instrument, making it unfavorable for long-term remote-controlled operation.

To further examine these effects of gas load, and thus, baseline height, on oceanographic ΔO_2_:Ar determinations, we operated our MIMS system (cryo distillation always enabled) during two cruises where we intentionally changed the overall gas intake and source pressure, i.e., delivery to the detector. During the Arctic cruise, the pressures at the ionization source were in the upper 10^–7^ mbar range. For the SO cruise, valve adjustments were made, bringing the source pressure to the lower 10^–6^ mbar range (Table 1). The comparison between the two cruise data sets therefore enabled us to establish that the total amount of gas in the vacuum system imposes notable effects on overall signal heights and baseline contributions: Raw signal intensities of all analytes increased by ca. 50% in the data from the SO cruise (Figure 6e,f and Table 1), even though true (calculated/measured) gas concentrations were lower than during the Arctic cruise. Concomitantly, the increased gas load raised the instrument baselines to ca. 76 and 73% of the 21/1% (air equilibrium) calibration signals of O_2_ and Ar, in contrast to only ca. 29 and 25% in the Arctic cruise with low gas load at the source (Figure 6e,f). As a consequence, the overall dynamic range of measurements was decreased by approximately 33% in the SO data as seen by the baseline corrected traces (Figure 6g,h). This lowered the instrument sensitivity to 0.3–0.5 μmol kg^–1^ for O_2_ (comparable to the sensitivity of manual Winkler titrations for O_2_) and 0.025–0.065 for Ar, which was also reflected by the approximately 50% higher calibration factors during the SO cruise compared to the Arctic cruise (Figure 6i,j and Table 1), Notably, over the duration of the Arctic cruise, overall baseline drifts of ca. −1 and −0.09 μmol kg^–1^ day^–1^ were observed for O_2_ and Ar, respectively, while during the SO cruise, baseline drifts were ca. −5 and −0.5 μmol kg^–1^ day^–1^. These drifts reflect the ongoing evacuation of the vacuum system. Under low pressure, gas molecules obey “molecular flow”, in which their movement does not follow a pressure gradient, but rather follows stochastic movement with straight trajectories. In vacuum systems with increasingly complex architecture (i.e., many corners, dead ends with sensor placements, corrugated steel tubings), water molecules can be trapped in niches for a long time at comparably low pressure, and their removal can take very long. This drift phenomenon further emphasizes the need to assess baselines frequently over the time of instrument operation, especially if the system was vented and initially had a high water background.

The “moving” ratio calibrators (Figure 6i,j) obtained using both, the uncorrected as well as the baseline-corrected approach (eqs 4 and 5) were very stable with precisions of 0.04% in ratio determination that are typically achievable also with other mass spectrometers.^47^ Thus, both pressure settings appear overall principally usable, as long as calibrations are done to account for baselines. Determined O_2_ concentrations (Figure 7a,b) are in both cruises overall in good agreement with independent determinations (e.g., CTD-mounted sensors), but short-term fluctuations in membrane and seawater temperature can affect the steadiness and reliability of the measurements between calibrations. Therefore, additional continuously recorded O_2_ sensor data is certainly a desirable amendment to MIMS data.

Accounting for baselines and using the two-point calibration approach substantially affected the calculation of ΔO_2_:Ar ratios. Specifically, during both cruises, the conventionally obtained ΔO_2_:Ar_(1R)_ was generally lower and exhibited compressed dynamics compared to the ΔO_2_:Ar_(2R)_ and ΔO_2_:Ar_(2I)_ approaches that include baseline determinations (Figure 7c,d). The high similarity between the latter two approaches derives from the fact that they share the same data basis (i.e., baseline-corrected data) but one is afflicted with minute uncertainties from the calculation of O_2_ and Ar solubilities, which yet tend to get larger at lower temperatures.^9,10^

Both phenomena observed in the ΔO_2_:Ar_(1R)_ data, i.e., lower values and compressed dynamics, seem to be mathematical artifacts derived from not accounting for the determined baselines, as can be seen from a simple case study (Table 2): Five scenarios are presented in which the unaccounted baselines in the O_2_ and Ar signals have a magnitude between 0% (i.e., no baseline at all) and 80% (i.e., high baseline contribution) of the 21/1% equilibrium calibration signal (row 1). Dividing the uncorrected (rows 2 and 4) or the corrected (rows 3 and 5) calibration signals can indeed yield the same calibration ratio (rows 6 and 7), often masking the problem. If a true excursion in M32 is observed (row 8), and baseline corrections are regarded (row 9), there will be a discrepancy between the uncorrected ratio (row 10) and the baseline-corrected ratio (row 11): The uncorrected approach will consistently detect an excursion of 5% supersaturation (row 12) while the baseline-corrected approach will detect excursions of up to 25% (row 13). In the case of a disregarded baseline of 80% calibration signal-height, this would mean an underestimation of the excursion by factor 5 (row 14).

**Table 2:** Case Study on Effects of Disregarded Baselines (See Text)

row	parameter	scenarios				
1	disregarded M32 and M40 baseline contribution to equilibrium signal (%)	0	20	40	60	80
2	exemplary observed M32 raw at calibration	1000	1000	1000	1000	1000
3	BL-corrected exemplary M32 at calibration	1000	800	600	400	200
4	exemplary observed M40 raw at calibration	50	50	50	50	50
5	BL-corrected exemplary M40 at calibration	50.0	40.0	30.0	20.0	10.0
6	M32:M40 raw ratio	**20**	**20**	**20**	**20**	**20**
7	M32:M40 BL-corrected ratio	**20**	**20**	**20**	**20**	**20**
8	assumed signal excursion in M32 raw signal (50 units)	1050	1050	1050	1050	1050
9	BL-corrected M32 with signal excursion	1050	850	650	450	250
10	M32:M40 raw ratio with excursion	21.0	21.0	21.0	21.0	21.0
11	M32:M40 BL-corrected ratio with excursion	21.0	21.3	21.7	22.5	25.0
12	“supersaturation” with neglected baseline	5%	5%	5%	5%	5%
13	“supersaturation” with accounted baseline	5%	6%	8%	13%	25%
14	underestimation factor due to baseline negligence	**1.00**	**1.25**	**1.67**	**2.50**	**5.00**

In other words, the higher the unaccounted baseline contribution, the stronger the resulting underestimation of excursions, which is the reason for the generally lower ΔO_2_:Ar values obtained when disregarding baselines, especially when operating at high source pressure and baselines, like in the SO cruise (Figure 7c,d). The fact that the underestimation of excursions is stronger when baselines are high therefore also explains the visibly stronger degree of compression of features in the ΔO_2_:Ar_1R_ data of the SO cruise, compared to the Arctic cruise (Figure 7c,d), where baselines had a low contribution to the observed signals.

Pressure, i.e., gas load, seems to be a main determinant of baseline height, but detector settings, source type, system architecture and membrane inlet are also likely to play a role. Therefore, the baseline height must be specific to an instrument and its current configuration. While there is not much published in the oceanographic literature about baselines, we investigated their existence in other instruments (Supporting Figure SF3): A Hiden RGA Quadrupole system connected to either an equilibrator inlet (EIMS) or a discoid silicone membrane inlet (MIMS) in the Kranz lab exhibited the lowest observed baseline contributions of <10% for O_2_ and Ar. Intermediary baseline contributions of 18–30% were observed for a Submersible Wet Inlet Mass Spectrometer (Transpector MPH quadrupole, Loose lab), a stable-isotope MS coupled to a discrete 8 mL cuvette (Micromass Isoprime, Rost lab), and a flow-through MIMS in an ecological observatory (Hiden HPR-40, Bowman Lab). Highest baseline contributions of 65% for O_2_ were observed in a Hiden pQA equipped with a direct probe inlet that was designed for high permeation and gas intake.

## Conclusion

Baseline determinations are critical elements of calibrations and are indispensable when accounting for instrument performance and drift over time. We show that gas load causes an increasing baseline contribution to the overall determined signal, and that disproportionate underestimations of the final ΔO_2_:Ar values will occur if such baselines are disregarded. Accordingly, published estimates of net community production where substantial instrument baselines were disregarded might be too low.

## Supplementary Material



## Notes

Flowchart of the data evaluation approach, exemplary calibrations from cruises as well as intercomparison of baseline contributions in various mass spectrometers (PDF)

## ○

Alfred-Wegener-Institute—Helmholtz-Centre for Polar and Marine Research, Am Handelshafen 12, 27570 Bremerhaven, Germany
