# Supplementary material for: Baselines Matter: Mass Spectrometric Assessments of Biological O2 Supersaturation (ΔO2:Ar) Benefit from Two-Point Calibrations
Source: Anal Chem. 2025 Nov 25;97(48):26364–73. doi: 10.1021/acs.analchem.5c02252 (PMC12874223; doi:10.1021/acs.analchem.5c02252)
Supplement: Supplementary file 1 [file ac5c02252_si_001.pdf]

Supporting Information

Baselines matter: Mass spectrometric assessments of biological O_2 supersaturation (ΔO_2:Ar) benefit from two-point calibrations

Sebastian D. Rokitta[*1], Emelia J. Chamberlain[2], Alessandra D´Angelo[3], Jeff S. Bowman[4], Brice Loose[3], Adam Ulfsbo[5], Allison A. Fong[1], Klaus-Uwe Richter[1], Sven A. Kranz[6] and Björn Rost[1,7]*

AFFILIATIONS

1: Alfred-Wegener-Institute – Helmholtz-Centre for Polar and Marine Research, 27570 Bremerhaven, Germany
2: Woods Hole Oceanographic Institution, Woods Hole, MA 02543, USA
3: University of Rhode Island, Graduate School of Oceanography, Kingston, RI 02881, USA
4: University of California San Diego, La Jolla, CA 92093, USA
5: University of Gothenburg, Department of Marine Sciences, 40530 Gothenburg, Sweden
6: Rice University, Houston, TX 77005, USA
7: University of Bremen, Department of Biology & Chemistry, 28359 Bremen, Germany

Pages: 4

Figures: 3

Table of contents:

Supplementary Figure SF1; Flow chart of our MIMS approach

Supplementary Figure SF2, a-f; Exemplary calibrations during PS122 and PS133

Supplementary Figure SF3, a-f; Baseline investigations in other instruments

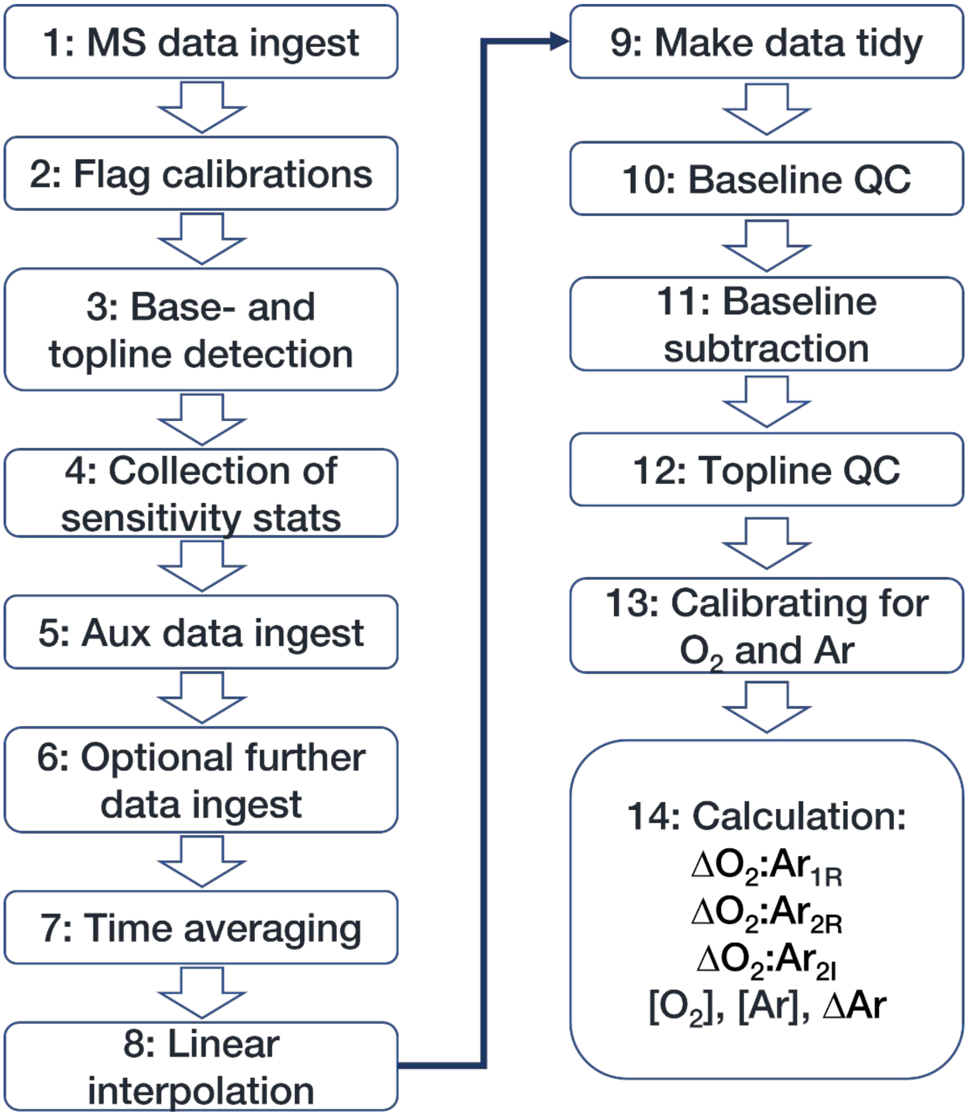

Supplementary Figure SF1: Flow chart of our MIMS approach. Raw data is ingested into R (1) and time ranges of calibrations are flagged (2). Baselines and toplines are detected algorithmically and with visual confirmation for every calibration (3). Instrument sensitivity and response times are determined (4) for every calibration. Then, auxiliary data (e.g., from ship´s sensors, 5) as well as further complementing data (6) are ingested, before data is time-averaged (7) and gaps are linearly interpolated (8). Data needs to be tidy, i.e., gapless (9), before baseline points are quality checked (10) and a moving baseline can be constructed and subtracted (11). Then, the determined 21%/1%, i.e., 'topline' calibration signals are quality checked based on their numeric value and the resulting O_2:Ar ratio (12), and moving calibrators for O_2 and Ar are calculated (13). Lastly, parameters of interest like ΔO_2:Ar ratios, concentrations of O_2 and Ar, as well as Ar supersaturation can be calculated (14).

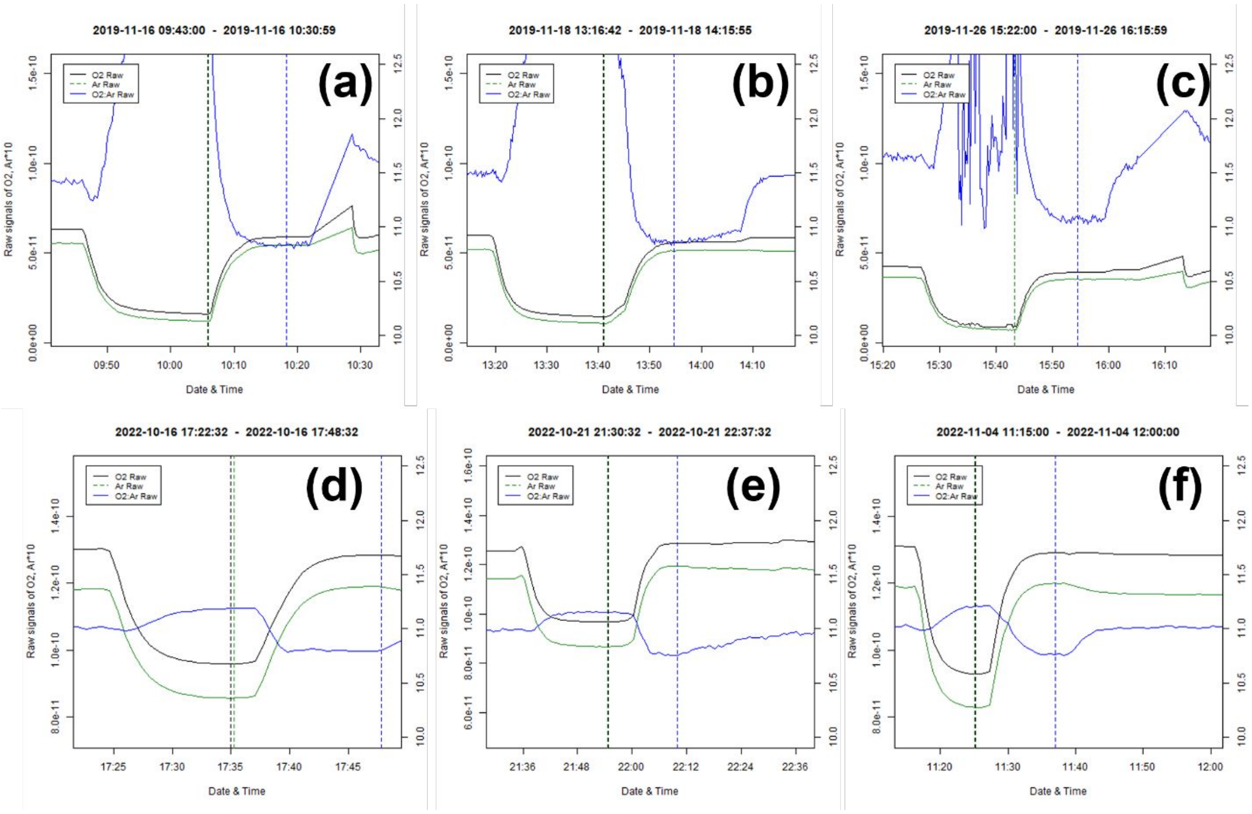

Supplementary Figure 2: Baselines obtained during the Arctic PS122 cruise (a-c) and the Southern Oceam cruise (d-f). Grey and green dashed lines indicate the 0% calibrations for O_2 and Ar, blue dashed line indicates 21/1% calibration points.

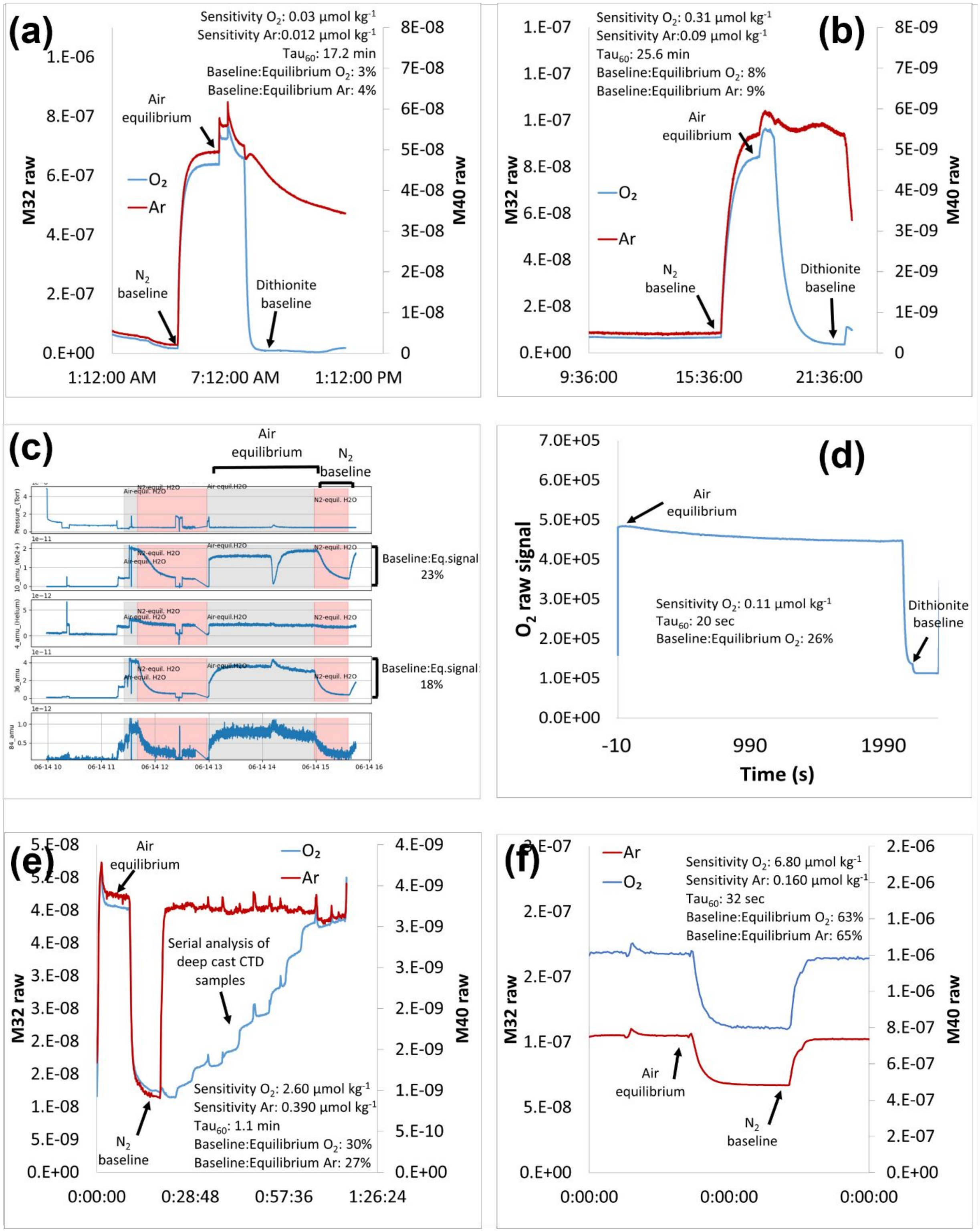

Supplementary Figure SF3: Calibrations and performance assessments in different instruments; a: EIMS (Kranz Lab); b: MIMS (Kranz Lab); c: Underwater MIMS (Loose Lab); d: discrete MIMS (Rost Lab), e: MIMS (Bowman Lab); f: Hiden pQA demo instrument. All calibrations show that baseline levels can contribute between 3 and 65% to the total equilibrium signal.
